# Regeneration of TiO_2_ Nanotube Arrays after Long-Term Cell and Tissue Culture for Multiple Use – an Environmental Scanning Electron Microscopy (ESEM) Survey of Adult Pig Retina and beyond

**DOI:** 10.1186/s12575-019-0090-4

**Published:** 2019-01-29

**Authors:** Sabrina Friebe, Stefan G. Mayr

**Affiliations:** 1 Leibniz-Institut für Oberflächenmodifizierung e.V. (IOM), Permoserstr. 15, 04318 Leipzig, Germany; 2 Division of Surface Physics, University of Leipzig, Linnéstraße. 5, 04103 Leipzig, Germany

**Keywords:** Long-term tissue culture, Neuronal tissue, Biomimetic surfaces, TiO_2_ nanotube arrays

## Abstract

Long-term organotypic culture of adult tissues not only open up possibilities for studying complex structures of explants in vitro, but also can be employed e.g. to investigate pathological changes, their fingerprints on tissue mechanics, as well as the effectiveness of drugs. While conventional culture methods do not allow for survival times of more than a few days, we have demonstrated recently that TiO_2_ nanotube arrays allow to maintain integrity of numerous tissues, including retina, brain, spline and tonsils, for as long as 2 weeks in vitro. A mystery in culturing has been the interaction of tissue with these substrates, which is also reflected by tissue debris after liftoff. As the latter reveals fingerprints of tissue adhesion and impedes with nanotube array reuse, we address within the present environmental scanning electron study debris nature and the effectiveness of cleaning approaches of distinct physical and chemical methods, including UV-light irradiation, O2 plasma treatment and application of an enzyme-based buffer. This will lays the foundation for large-scale regeneration and reuse of nanotube arrays in science and clinical research.

**Electronic supplementary material:**

The online version of this article (10.1186/s12575-019-0090-4) contains supplementary material, which is available to authorized users.

## Background

Nanotubes of different materials have attracted tremendous scientific interest during the past two decades due to their high versatility for a variety of applications [1, 2]. Depending on their chemical and mechanical properties they can be used, e.g. as carrier material in catalysts or for drug delivery [3]. Most prominently, their surface structure reveals a well-ordered, highly porous morphology originating from a grid-like alignment of nanotubes with highly tunable diameter, wall width and roughness, as conveniently controlled by the synthesis parameters [4, 5]. While TiO_2_ nanotube arrays, that have been synthesized via electrochemical anodization, have been found suitable for cell culturing and stem cell differentiation [6–8], our work is focused on long-term organotypic cultivation of adult tissue [9], as demonstrated by developing scaffolds with highly optimized nanotube geometries for retina [10], brain (neocortex, hippocampus), spleen and tonsils [11]. Due to their mechanical stability and morphological adjustability, they have been found particularly useful for biomechanical tissue assessments [12], filling the gap between standard cell culture and in vivo studies [10, 11, 13]. Furthermore, surface morphology and roughness as well as physical properties like surface charges and surface free energy [14] have critical influences on cell or tissue adhesion to the substrate and serve as important conditions for integrity and preservation of tissue architecture, cell adhesion and motility [6, 8, 15–17]. Thus, it was possible to measure mechanical properties of small and sensitive tissues as demonstrated by Rahman et al. [12]. However, despite that TiO_2_ nanotubes are used as a tool for cleaning, e.g. the separation of oily substances from water or as photocatalytic active membrane, little is known about cleaning TiO_2_ nanotubes themselves from biological residues for reuse, after employing them as culture substrates [18, 19].

Due to the excellent biocompatibility, titanium is an often used implant material in medicine. It is well known how to sterilize implant surfaces with distinct methods like UV-light or O_2_-plasma [20–22]. Indeed, these methods are partially suitable to clean the TiO_2_ nanotubes surface from biological contamination as we will show. For qualitative analysis we used an environmental SEM to investigate the cleaning characteristics of treatments with UV-light, O_2_-plasma and proteinase K.

## Results

### Cell Proliferation on Nanotube Arrays

**Fig. 1:**
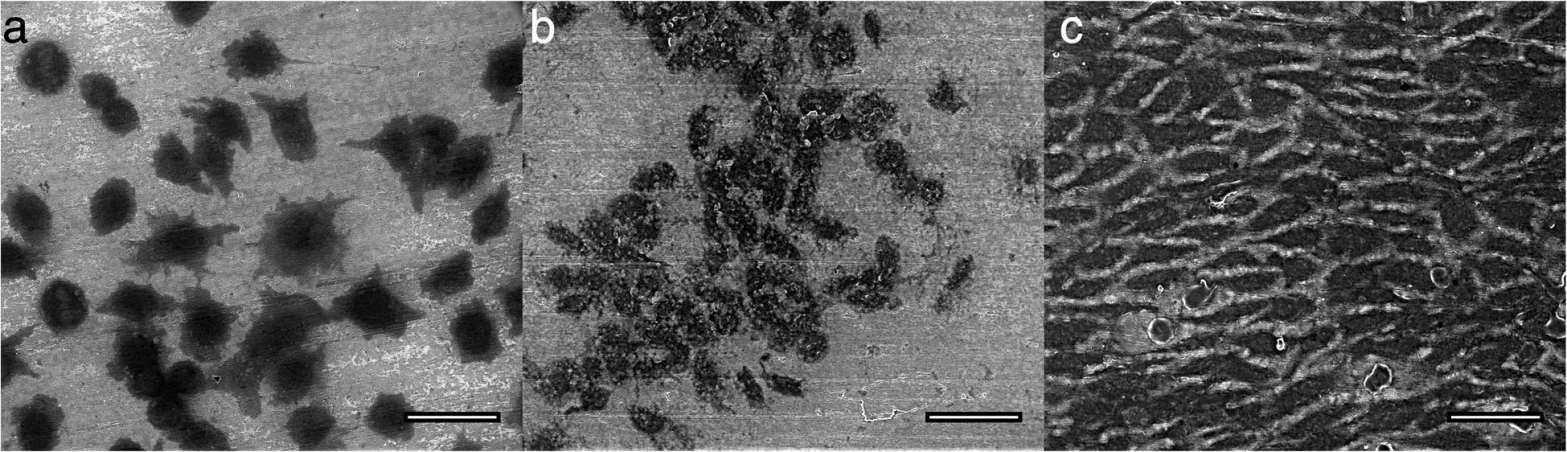
ESEM images of L929 mouse fibroblast cultured on top of customized nanotube arrays with a tube diameter of (32 ± 3) nm for different cultivation times: 1 day **(a)**, 4 days **(b)** and 7 days **(c)**. Scale bars correspond to 50 μm

In a first step we employed mouse fibroblasts (cell line L929) that were cultured for 7 days on specifically tuned nanotube arrays tube diameter (32 ± 3) nm) as model system to address within an environmental scanning electron microscope (ESEM) study cell adherence, lift-off and nanotube regeneration. In fact, the L929 cell line is widely used and due to their easy handling we can ensure almost identical nanotube-cell-samples for investigations of different nanotube regeneration approaches in a well-controllable and reproducible manner. Integrity of cells on top of the nanotube arrays was characterized using the ESEM and compared to cells in culture dishes as reference (not shown here). Both culture substrates have the same growth area of 1cm^2^. After 1, 4 and 7 days, nanotube samples were imaged with the ESEM, as shown in Fig. 1. Within 24 h, cells are well stretched, not rounded up and adhered well to the nanotube array, as can be concluded from formation of lamellipodia. After 4 days in culture, cells arrange in groups and form an aggregate which makes it difficult to distinguish individual cells from each other. With increasing density, cells lose their stretched shape and become more spherical, overlap and grow over each other. On top of nanotube substrates they reach confluency after 7 days indicating a doubling time of 38 h. Compared to cells grown in standard culture wells which show a doubling time of 22 h, L929 cells grow significantly slower on nanostructured surfaces. As soon as characterization using ESEM was finished, cells were fixed with 4% PFA for 24 h and dried overnight in a vacuum-assisted furnace.

### Adult Porcine Retina Explants

Successful long-term culture of guinea pig adult retinal tissue is one of the key achievements that became feasible by introduction of nanotube substrates as reported by us previously [10]. While conventional culture methods failed to maintain tissue integrity, nanotube arrays can be tuned for different cell and tissue types to enable long-term organotypic culture of adult tissues [7, 10]. Until now, however, it has not been unveiled how tissue interacts with the surface of nanotube arrays and, in particular, how adhesion is mediated. Residuals after tissue lift-off and their removal from the surface of the nanotube arrays reveal a fingerprint of adhesion. Vice versa, from a practical point of view, residual removal opens up the possibility of nanotube regeneration and reuse after tissue culture. For that purpose we synthesized nanotubes with a diameter of d = (72 ± 3) nm which are proven suitable for long-term culture of adult guinea pig retinal tissue [10]. In contrast to previous studies, however, we presently employed retina explants from pig eyes provided by a slaughterhouse. This novel approach is motivated by their larger similarity to human eyes due to vascularization of retinal tissue and presence of macula, when compared to commonly studied rabbit or guinea pig eyes. In fact, by demonstrating successful organotypic culture of porcine retina explants from slaughterhouse animals on reusable nanotube arrays, we pave the way for a highly sustainable novel in-vitro assay for in-depth biomedical studies completely without the need for lab animals.

**Fig. 2:**
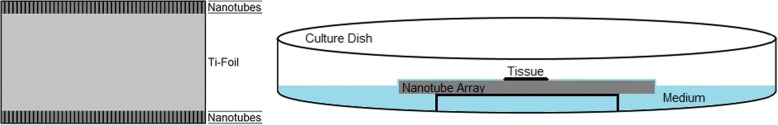
Structure of a TiO_2_ nanotube array with about 10 μm TiO_2_ nanotubes at the top and the bottom of the Ti-foil (left) and a schematic image of the tissue culture setup (right), where a retina explant (black) lies on top of nanotube array (grey) which is placed onto a stainless steel grid (black). Super-hydrophilicity ensures a thin medium film (blue) on top of the arrays and supplies the explant with needed nutrients

In doing so, retinae were extracted from the eye-ball and cultured with photo receptor side down on top of nanotube array. A stainless steel grid is put into a petri dish and nanotube array is placed on top of the grid. Medium was filled up so that it came into contact with underside of nanotube array but did not cover the top of them. Due to super-hydrophilicity of TiO_2_ nanotube arrays, medium flows from bottom to top over edge of the nanotube array, ensuring a thin medium wetting layer on top and enables at the same time free gas exchange with the environment, as visualized in Fig. 2.

**Fig. 3:**
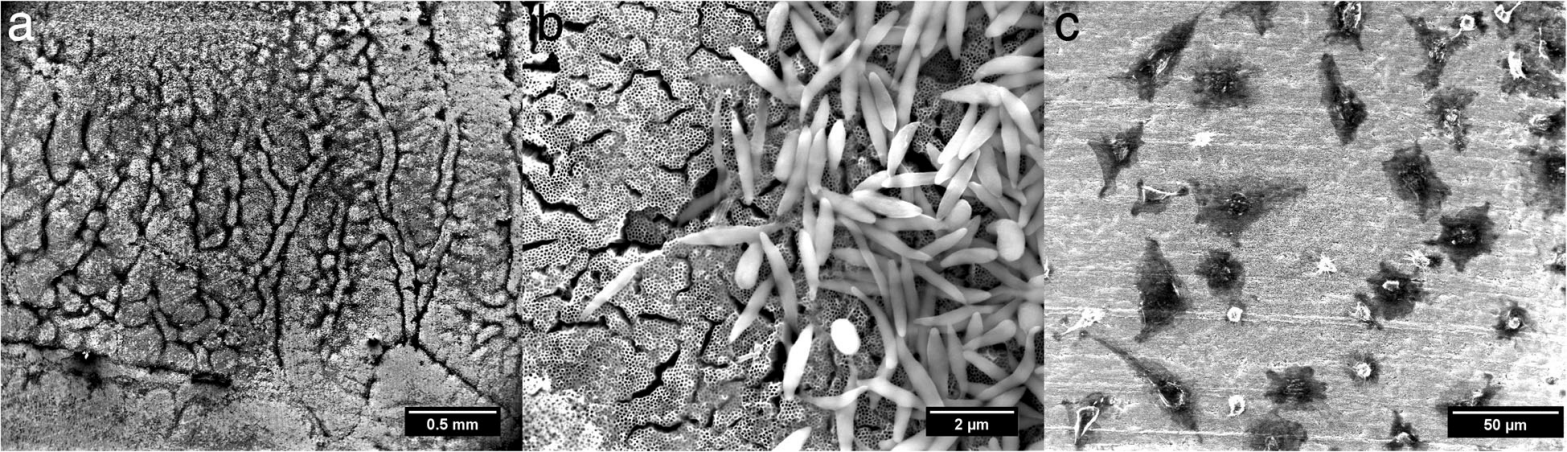
ESEM images of PFA/GA-fixed retinal tissue residues on top of nanotube arrays after 14 days in culture: (**a**) overall view for retinal residues (**b**) microvilli from the RPE and (**c**) out-migrating cells which collect at the edges of the scaffold

After incubating at 37 °C and 5% CO_2_ for 14 days, retina was fixed on top of nanotubes with mixture of 2% glutaraldehyde and 2% paraformaldehyde for 24 h and carefully removed from nanotube array using a brush and pipette. Nanotube arrays with retina residues on top were investigated using the ESEM. As shown in Fig. 3, we can identify different parts of retina, like cells from the retinal pigment epithelium (RPE) and their microvilli. Visualization of rods and cones between remaining retinal tissues using the ESEM was not possible due to disrupted residues. However, we could confirm presence of rods and cones on top of the surface with the help of a fluorescently staining. Figure 3a shows the residuals after removal of cultured retinal tissue, which reflects a highly heterogeneous nature with regions highly populated with and vacant of residues, respectively. In some cases we found cells that migrated from the injured tissue edges towards the not used substrate periphery Fig. 3c. In fact, out-migration rate is a good indicator for degeneration processes within the tissue and in future studies it should serve as another indicator for a successful cultivation.

### Residue Removal and Nanotube Regeneration

**Fig. 4:**
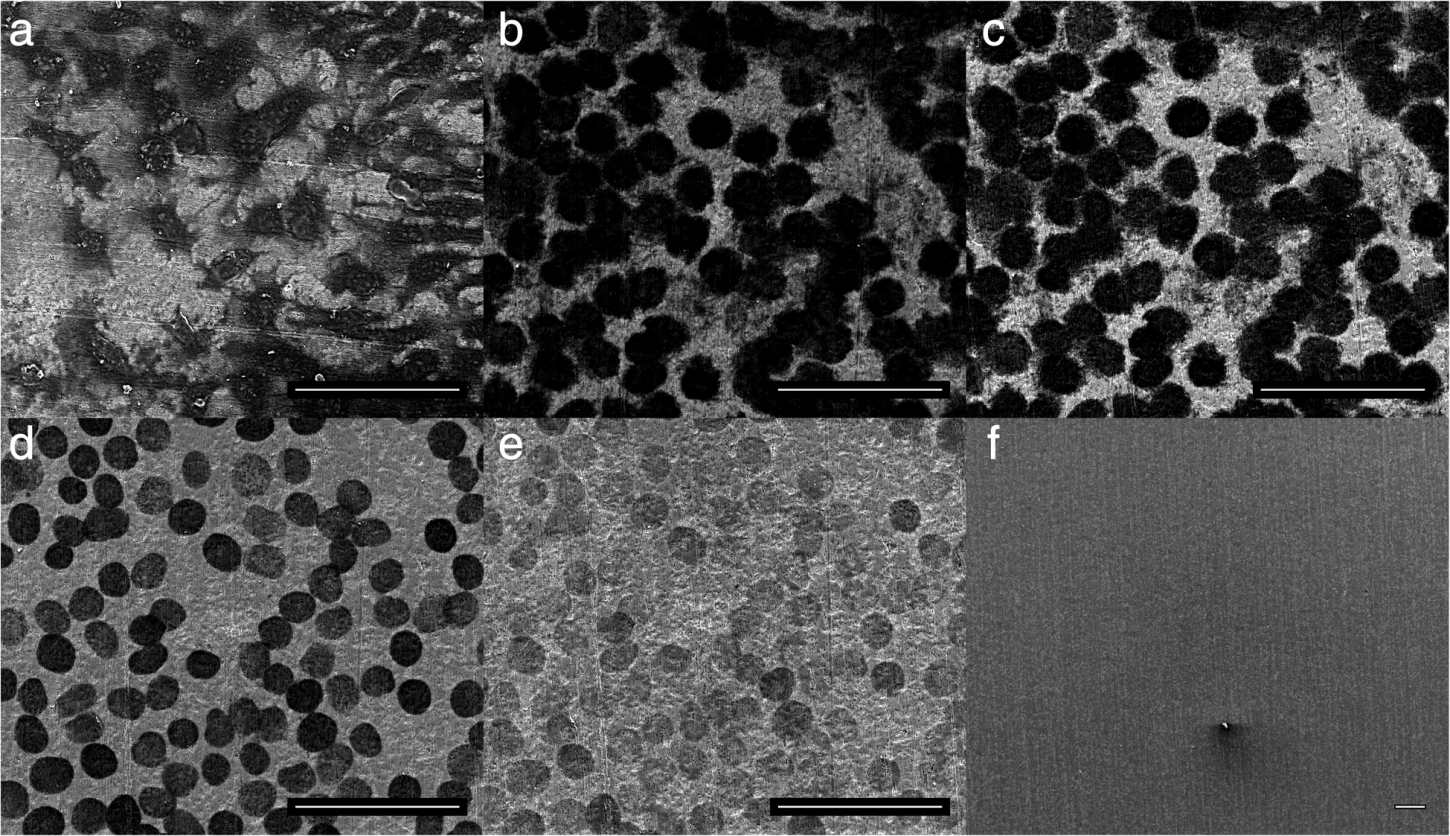
ESEM image of living cells on top of a nanotube array (**a**) and SEM images for different UV-light irradiation times on PFA fixed cells: 0 min (**b**), 2 min (**c**), 22 min (**d**), 62 min (**e**) and 102 min (**f**). Scale bars correspond to 50 μm

Irradiation with UV-light was one of two successful methods to regenerate nanotube surfaces partially or completely by removing single cell residues. For a systematic assessment we cultured L929 cells on top of nanotube arrays for 7 days, checked doubling time and adherence to ensure viability and a homogeneous cell layer to mimic a tissue layer, respectively. Figure 4a shows L929 cells at the peripheral region of a nanotube array after 1 week culturing. While the cell layer in the periphery was not completely closed, it was purposely chosen as it makes it easier to find the same position during imaging for direct qualification of cleaning progress during UV light treatment. After cultivation, samples were imaged with ESEM to check residues (top Fig. 1c), PFA-fixed and dried in a furnace overnight. Afterwards samples were placed inside of the UV-light irradiation chamber and irradiated for overall t = 102 min with a wavelength of λ = 172 nm. To visualize cleaning progress by UV-light exposure, we used one sample for imaging at different stages of UV-light cleaning. First, untreated and living cells were imaged (before PFA-fixation) in ESEM-mode serving as control for cell density and integrity (Fig. 4a). Right after that, the samples were PFA-fixed, dried overnight and imaged in high-vacuum-mode after different UV-light exposure times at the same position for 0 min, 2 min, 22 min, 62 min and 102 min, as shown in Fig. 4b-f, respectively. Using ESEM, we confirmed that the surface was becoming cleaner with increased duration of UV light treatment without damage of the nanotube array.

Exposure of contaminated nanotube arrays to O_2_-plasma constitutes a highly promising alternative, as supply of reactive oxygen is expected to react instantaneously with cell residues to gaseous CO_2_, resulting in a clean surface.

**Fig. 5:**
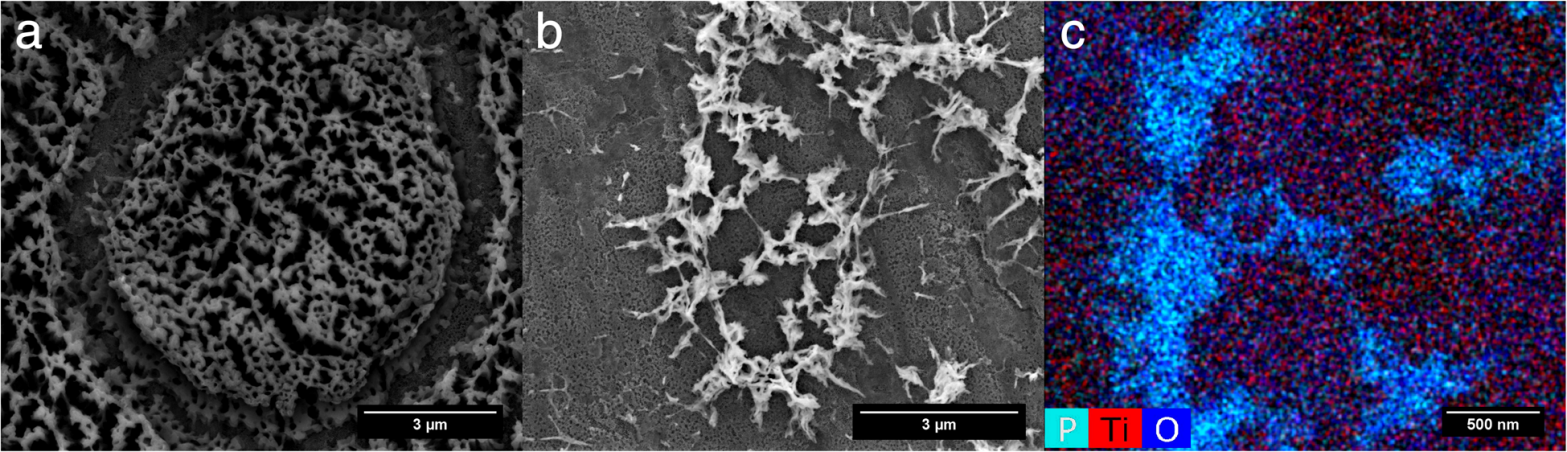
ESEM image of O_2_-plasma treated cell residues after 5 min (**a**) and 30 min (**b**) and an EDX-analysis of remaining filament-like residues after 30 min (**c**) which shows that the debris are made of phosphorous (P) and oxygen (O) laying directly on top of the TiO_2_-nanotube surface (Ti + O)

Just as for UV-light irradiation, samples were PFA-fixed, dried in furnace overnight and imaged with ESEM serving as control and baseline for comparison between cleaning steps. Nanotube arrays with cell residues were placed inside the plasma chamber which was evacuated and flushed with oxygen. Samples were treated for t = 45 min total; after 5 min O_2_-plasma treatment, cell membrane and surrounding matrix got broken apart, while after completion of treatment (30 min) only filamentous-like residues were left (Fig. 5a-b). Further continuation of the O_2_-plasma treatment did not result in significant decrease of these filaments; images taken after 45 min O_2_-plasma treatment pretty much look the same as after only 30 min. Analysis using energy dispersive X-ray spectroscopy (EDX) within our ESEM unveiled their chemical nature, viz. high oxygen and phosphorous content (Fig. 5c).

To track down the origin of the cell residues observed after O_2_-plasma treatment, we considered three possible origins of the residues, viz- DNA, adhesion proteins and membrane residues, which we incubated individually for 24 h each on top of nanotube arrays and imaged cleaning progress during O_2_ plasma treatment within our ESEM. Only the residues observed for FCS, which contains ample adhesion proteins, comply with the cell-nanotube samples in terms of patterns and chemical composition, while particularly DNA isolated from our cell line did not. This lead us to the conclusion, that the phosphorous deposits after O_2_ plasma treatment originate from adhesion proteins expressed by the cells.

**Fig. 6:**
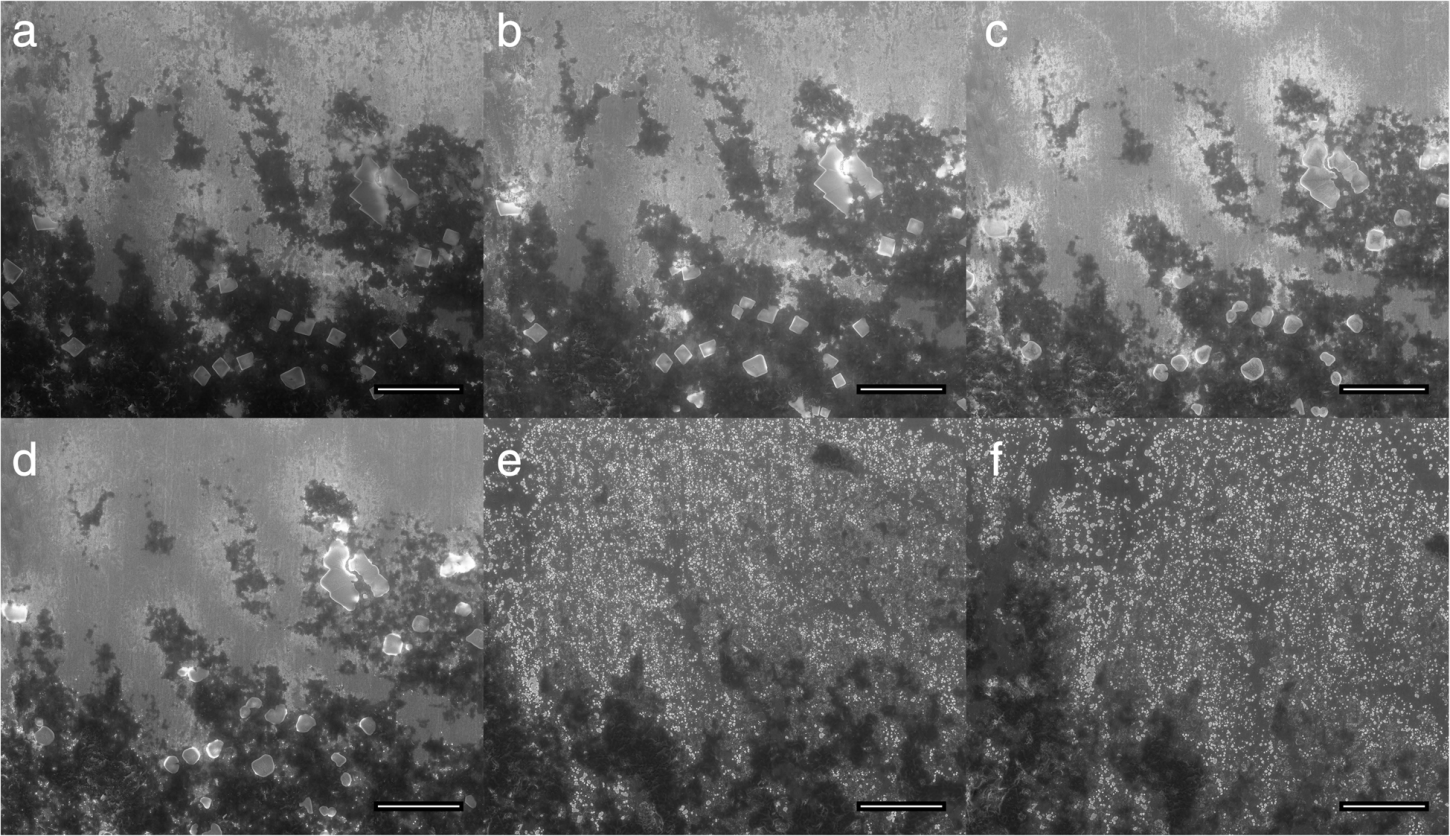
ESEM images of tissue residues on top a nanotubes array for step by step UV-light irradiation imaged at distinct times: 0 h, 1 h, 2 h, 3 h, 7 h and 15 h (**a**-**f**), respectively. Scale bars correspond to 50 μm

It turned out that cleaning of nanotubes surfaces from tissue residues is much more complex than for single cells. Exposure to UV-light, that was highly successful for individual cells, turned out to be a lengthy procedure for removal of tissue residues with were partially still present for exposure times as long as 15 h. While we expect full removal of residuals for sufficiently large times, we thus regard this approach unsuitable from a practical point of view. Figure 6 shows a cleaning approach that was performed in direct correspondence to our cell experiments.

In fact, tissue residues were partially removed, but it takes much more time as for the simplified cell culture model. Furthermore, we see an increase in free salt deposits with increasing UV exposure time.

**Fig. 7:**
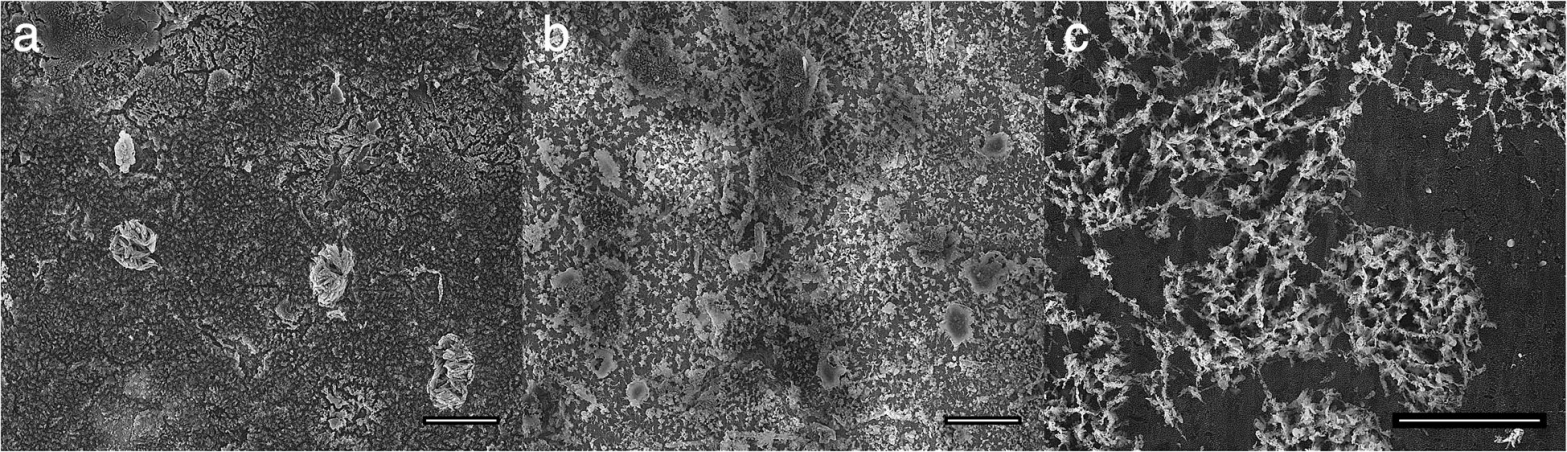
Distinct cleaning steps of tissue residues using O_2_-plasma treatment for 25 min (**a**), 85 min (**b**) and 145 min (**c**) respectively. Scale bars correspond to 10 μm

As for cleaning efficiency of O_2_-plasma treatment to remove retinal tissue residues, Fig. 7 reveals that while the cell membrane got ripped apart, after 145 min filamentous-like deposits originating from adhesion proteins which again cannot be removed by O_2_-plasma treatment, rendering it useless for cleaning purposes.

Our final approach to employ a mixture of lysis buffer with proteinase K turned out to be the method of choice to fully remove retinal tissue residuals and thus regenerate nanotube arrays for reuse. While we have employed this method before [11], its efficiency to fully remove all debris from the nanotube array is systematically assessed here for the first time. Nanotube substrates with tissue residues on top were incubated in lysis buffer (0.5 mg/ml proteinase K) for 24 h at 55 °C overnight in furnace. After enzymatic treatment, no traces of tissue residues on the nanotube surface could be detected (see Additional file 1), leaving also the nanotube surface intact and indistinguishable from the as-synthesized nanotube arrays. It thus constitutes the method of choice for regeneration of nanotube arrays after retinal tissue culture, and presumably beyond.

## Discussion

Long-term tissue culture of adult retinal tissue on top of custom-synthesized nanotube substrates should close the gap between cell culture and in vivo studies, especially in terms of structure and function preservation, as well as enhancement of the cultivation time for further studies of neurodegenerative diseases and drug screening. Thus, cleaning and reusability of these arrays should also be in focus of research for an economic and resource saving work.

In our study, L929 cell line and retinal tissue (originally isolated from pigs) were cultured on top of TiO_2_ nanotube arrays for the first time and investigated using environmental scanning electron microscopy with regard to their cleaning characteristics by using UV-light and O_2_-plasma exposure, as well as enzymatic treatment. We identified significant differences for both cleaning methods and cultivated biological material. To ensure cell and tissue survival, free and single standing nanotubes with a tube diameter of (32 ± 3) nm and (72 ± 3) nm were used for single cell cultivation [7] and retinal tissue cultivation [10], respectively. We observed good adhesion of cells and tissue on the arrays, as it was shown in previous studies [7, 10, 11].

Before cleaning methods were initiated we studied cell- and tissue-nanotube-interfaces using an ESEM to control adhesion of single cells and tissue to nanostructured surfaces. For the L929 cell line, we investigated adhesion and doubling time for reasons of proliferation and to ensure that cells are able to form a continuous and closed layer serving as simplified model of tissue at which cleaning methods can be tested. We found good cell adhesion to arrays within 24 h and a smaller doubling time as compared to cells grown in culture dishes which is due to the improved adhesion on nanotube substrates. A homogenous and closed cell layer was observed after 7 days in culture. For tissue cultivation we checked adhesion as well, by cultivating retina for more than 7 days, removing it from the nanostructures surface and investigating the remaining residues using ESEM. Just like for single cell culture, we found good adhesion after 24 h and a continuous adhesion border for tissue to substrate.

We performed a number of investigations focusing on different protocols for residue removal from nanotube arrays after application in cell and tissue culture (data not shown here). Due to general usage of trypsin in cell culture, it was employed in a first approach for regeneration of nanotube surfaces. As even after treatment for 24 h with trypsin/EDTA at humid atmosphere no or only little cleaning effects were observable, we conclude that a simple trypsin treatment was not sufficient for cleaning the nanotubes. Other investigations like enzymatic treatment with dispase, treatment in an ultrasonic bath or combination of all mentioned methods did not lead to desired results either and thus will not be discussed here.

As first cleaning method UV-light irradiation was tested. It is generally known that wavelengths below 200 nm are able to split the oxygen compounds into atomic oxygen and ozone simultaneously [20]. In our experiments with single cells, it is shown that the nanotube surface is completely cleaned within 100 min so a reuse of these scaffolds in cell culture is possible in contrast to UV-light irradiated tissue residues. Therefore, we demonstrate that the UV-light works, but the effect is much slower due to the thickness of the remaining residues and also unknown substances preventing the dissociation and preserving the molecular structure making this an unsuitable treatment method.

The second cleaning approach employed O_2_-plasma, which basically works much faster than UV-light irradiation but suffers from filamentous-like phosphate deposits for single cell and tissue residues.

A complete tissue residues cleaning without using physical treatments is achieved using a lysis buffer with Proteinase K within 24 h. Here, retina residues were removed from the nanotube arrays without damaging the nanotube structure. When nanotube arrays were cleaned with this approach, the arrays looks exactly like their freshly produced counterparts in ESEM measurements, as well as the cleaned surface from picture 3f, making them indistinguishable from the as-synthesized substrates.

When comparing synthesis and cleaning of the nanotube arrays in terms of required economical and human resources, a significant advantage of cleaning can clearly be confirmed: While synthesis of new arrays causes ten times as high costs for consumables as cleaning using lysis buffer with Proteinase K (excluding personnel expenses), the time scale required for cleaning nanotube arrays is only half as large as for a new synthesis. Apart from that, we would like to emphasize that production is much more complex than the cleaning procedure, which can be realized virtually by every user of the nanotube substrates.

## Conclusion

In conclusion, recent studies show that adhesion to underlying substrate is important for a successfully tissue culture and our novel biotechnological concept of TiO_2_ nanotube arrays offer that possibility. We demonstrate how to clean such arrays for a further usage as cultivation substrate. In summary, we found that the cleaning method of choice is determined by the cultured biological material. For classical cell culture, UV-light irradiation is sufficient to clean the whole nanotube surface, whereas for tissue cultivation on top of nanostructured arrays a proteinase-K-based lysis buffer has to be used. Thus, it is possible to reuse customized TiO_2_ nanotube arrays for several cultures for saving resources and work economically.

## Materials and Methods

### Production of TiO_2_ Nanotubes

TiO_2_ nanotubes were produced by electrochemical anodization at room temperature. In doing so, titanium foil (Advent Research Materials Ltd., 0.1 mm thickness, 99.6 + % purity) was first cut in the desired size, for our experiments with retinal tissue 20 mm × 30 mm, cleaned by ultrasonication each for 10 min in distilled water and isopropyl alcohol and dried in a nitrogen stream. Cut and cleaned titanium foil served as anode and a platinum-mesh as cathode. Both electrodes were fixed with a constant distance of 45 mm. Titanium-foil was chemically etched by immersing both electrodes into electrolyte (consisting of 98% ethylene glycol, 2% distilled water and 0.3% ammonium fluoride powder) and anodized for 60 min. For experiments with the murine L929 cell line, size of scaffolds was chosen as 10 mm × 10 mm. An anodization voltage of 16 V was used leading to nanotubes with a diameter of d = (32 ± 3) nm, which were suitable for single cell culturing [7]. For experiments with retinal tissue an anodization voltage of 50 V was used which leads to a tube diameter of d = (72 ± 3) nm [10]. Each anodized TiO_2_ nanotube array was cleaned with ethylene glycole, gently dried with nitrogen stream and afterwards in furnace at 42 °C overnight. To complete nanotube production and to make them usable for single cell culturing, a final cleaning step was performed in an ultrasonic bath with distilled water for 10°min to ensure a complete removal of all electrolyte residues.

### Cultivation of L929 Mouse Fibroblasts

Each nanotube array (1 cm^2^ nanotube surface) was synthesized and cleaned as described above, dried and set into a petri dish. First, cells were grown in a 75 cm^2^ cell culture flask and passaged when they reached a confluency of 80%. 10,000 cells were applied to the middle of the nanotube array and into each well of a 48-well plate (1 cm^2^ growth area). Cells were allowed to settle down and adhered on nanotube surface for 2 h in incubator at 37 °C, 100% humidity and 5% CO_2_. Subsequently, 1.5 ml (48 well-plate) and 3 ml (nanotube array) culture medium were added and samples were cultured at most 7 days while changing culture medium every third day. Standard culture medium (90% RPMI with 10% fetal calf serum and 0.1% gentamycin) was utilized.

Using ImageJ, cell numbers were calculated as measure of doubling time. Images of cells grown in culture dishes and on nanotube surfaces were taken every day. When they became denser and grew over each other it was not possible to distinguished single cells from each other; therefore a further comparison between confluency was performed. Using *N* = *N*_0_*e*^*kt*^ the doubling time can be calculated.

### Cultivation of Adult Pig Retina on Top of Nanotube Arrays

Pig eyes were obtained by courtesy of “Emil Färber GmbH Großschlächterei & Co. KG, Belgern-Schildau”. Pigs were on average 6 to 7 months old and weighted 100-150 kg. They were stunned individually by electric shock and bleeding to death. Right after, eyes were removed with 3–5 mm optical nerve by a local butcher and placed in chilled phosphate-buffered saline (PBS) at a temperature of 4 °C. Eye transport from slaughterhouse to laboratory started immediately and did not last longer than 2 h. The eyeball was opened with scalpel and scissors and the vitreous body was disconnected from retina under sterile conditions. Carefully, the retina was separated from retinal pigment epithelium (RPE) using tweezers, cut in desired size (about 0.8 cm^2^) and placed with photoreceptor side down on top of a nanotube array which itself is placed on top of a stainless steel grid (l x w x h: 30 mm × 30 mm × 5 mm) inside a petri dish. Medium (Ames Medium with 0.1% gentamycin and 10% horse serum) was added in such a way that it came into contact with nanotube array but did not cover it to guarantee gas exchange. The whole setup was incubated at 37 °C and 5% CO_2_ for 7 or 14 days. After cultivation, the retina was fixed with 2% glutaraldehyde (GA) (Serva, 23,115) and 2% paraformaldehyde (PFA) (Merck Millipore, 1.04005.1000) over night and removed from nanotube surface using a pipette and a commercial soft brush.

### Sample Preparation for ESEM Analysis

ESEM analyses were all carried out with a Quanta FEG scanning electron microscope (FEI Munich, Germany) which has three operating vacuum modes: high vacuum, low vacuum and ESEM (environmental). Furthermore, an EDX (energy dispersive X-ray) spectroscope is included in our ESEM setup. ESEM analyses were performed to investigate adhesion of individual cells grown on top of nanotube surfaces to ensure cell adherence and to check for remaining residues originating from tissue cultivation. After that, distinct cleaning methods were tested for retinal tissue residues as well. To analyze residues and cleaning effects, high vacuum mode was used for imaging. For ESEM-mode analyses, nanotube arrays with living cells on top were mounted into a special holder in which 4–5 droplets of distilled water were filled. Measurement started immediately and lasted up to 2 h maximum. Humidity and temperature were set to 40–70% and 4–8 °C, while pressure varied from 400 to 700 Pa. After completing ESEM-measurements of L929 cells cultured on top of nanotube arrays, samples were fixed with 4% PFA and some of them were stained for fluorescent microscopy while others were dried in furnace at 42 °C overnight for residual analysis. Samples were cleaned with UV-light or O_2_-plasma for different durations, as described in next chapter. ESEM analysis was continued without any washing steps in-between to make sure that cleaning effect came solely from the cleaning method. Cleaning procedure and measurements were repeated until no changes on residues could be detected or the surface was completely free of debris. The same preparation conditions as for single cells were applied for tissue residues on top of nanotube arrays: samples were fixed with 2% GA and 2% PFA, some samples were used for fluorescent microscopy to confirm presence of rods and cones while other samples were dried overnight and used for different cleaning procedures.

### UV-Light Irradiation, O_2_-Plasma and Proteinase K Treatment

For all samples, UV-light irradiation or O_2_-plasma treatment was carried out and intermittently interrupted for inspection of cleaning effects using ESEM. For treatment with UV-light, fixed and dried samples were inserted into the irradiation chamber which was flushed with dinitrogen after evacuation. A 172 nm/630 Z lamp system with a specified power of 16 mW cm^− 2^ was applied, choosing the distance between radiation source and scaffold as 26 mm. In total, a cell-nanotube sample was irradiated for 102 min, corresponding to a total irradiation dose of H = 96 J cm^− 2^. For tissue-nanotube samples, the UV-light treatment was terminated after 15 h.

For O_2_-plasma treatment, nanotube arrays were inserted into the plasma chamber which was flushed with oxygen and exposed to a 100% O_2_-plasma. Cell and tissue samples were treated with O_2_-plasma for 45 min and 145 min to remove cell and tissue residues, respectively.

For the enzyme-based cleaning method, 100 ml of a stock solution was synthesized (composed of 10 ml 100 mM Tris-HCL pH 8.5, 1 ml of 5 mM EDTA, 1 ml of 0.2% SDS, 4 ml of 200 mM NaCl and 84 ml Aqua-dest) and Proteinase K was added with a concentration of 0.5 mg/ml right before use. After incubation of nanotube arrays at 55 °C for 24 h in a furnace, the substrates were washed for several times with PBS.

## Additional file

Additional file 1: Treatment with lysis buffer and Proteinase K. ESEM images of nanotube surfaces before (a) and 24 h after the treatment with lysis buffer and Proteinase K (b). (PNG 2146 kb)

## Abbreviations

EDX: Energy-dispersive X-ray spectroscopy
ESEM: Environmental scanning electron microscope
GA: Glutaraldehyde
PFA: Paraformaldehyde
RPE: Retinal pigment epithelium
