# Supplementary figures and images for: Regeneration of TiO2 Nanotube Arrays after Long-Term Cell and Tissue Culture for Multiple Use – an Environmental Scanning Electron Microscopy (ESEM) Survey of Adult Pig Retina and beyond
Source: Biol Proced Online. 2019 Jan 29;21:2. doi: 10.1186/s12575-019-0090-4 (PMC6350358; doi:10.1186/s12575-019-0090-4)

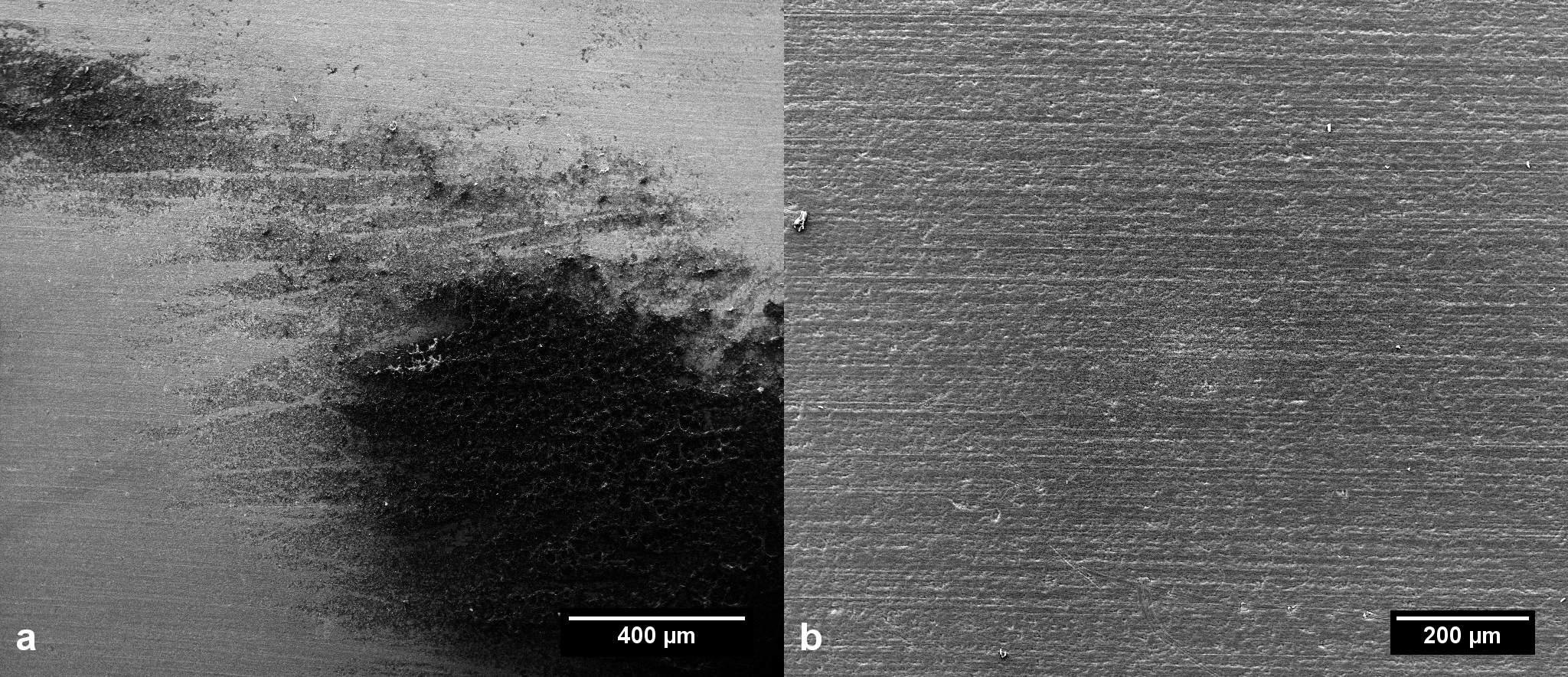

Supplement: Supplementary file 1 — Treatment with lysis buffer and Proteinase K. ESEM images of nanotube surfaces before (a) and 24 h after the treatment with lysis buffer and Proteinase K (b). (PNG 2146 kb) [file 12575_2019_90_MOESM1_ESM.png]
